# Identification and validation of potential prognostic lncRNA biomarkers for predicting survival in patients with multiple myeloma

**DOI:** 10.1186/s13046-015-0219-5

**Published:** 2015-09-11

**Authors:** Meng Zhou, Hengqiang Zhao, Zhenzhen Wang, Liang Cheng, Lei Yang, Hongbo Shi, Haixiu Yang, Jie Sun

**Affiliations:** College of Bioinformatics Science and Technology, Harbin Medical University, Harbin, 150081 PR China

**Keywords:** Biomarkers, Expression profile, Long non-coding RNAs, Multiple myeloma, Overall survival

## Abstract

**Background:**

Dysregulated long non-coding RNAs (lncRNAs) have been found to have oncogenic and/or tumor suppressive roles in the development and progression of cancer, implying their potentials as novel independent biomarkers for cancer diagnosis and prognosis. However, the prognostic significance of expression profile-based lncRNA signature for outcome prediction in patients with multiple myeloma (MM) has not yet been investigated.

**Methods:**

LncRNA expression profiles of a large cohort of patients with MM were obtained and analyzed by repurposing the publically available microarray data. An lncRNA-focus risk score model was developed from the training dataset, and then validated in the testing and another two independent external datasets. The time-dependent receiver operating characteristic (ROC) curve was used to evaluate the prognostic performance for survival prediction. The biological function of prognostic lncRNAs was predicted using bioinformatics analysis.

**Results:**

Four lncRNAs were identified to be significantly associated with overall survival (OS) of patients with MM in the training dataset, and were combined to develop a four-lncRNA prognostic signature to stratify patients into high-risk and low-risk groups. Patients of training dataset in the high-risk group exhibited shorter OS than those in the low-risk group (HR = 2.718, 95 % CI = 1.937-3.815, p <0.001). The similar prognostic values of four-lncRNA signature were observed in the testing dataset, entire GSE24080 dataset and another two independent external datasets. Multivariate Cox regression and stratified analysis showed that the prognostic power of four-lncRNA signature was independent of clinical features, including serum beta 2-microglobulin (Sβ2M), serum albumin (ALB) and lactate dehydrogenase (LDH). ROC analysis also demonstrated the better performance for predicting 3-year OS. Functional enrichment analysis suggested that these four lncRNAs may be involved in known genetic and epigenetic events linked to MM.

**Conclusions:**

Our results demonstrated potential application of lncRNAs as novel independent biomarkers for diagnosis and prognosis in MM. These lncRNA biomarkers may contribute to the understanding of underlying molecular basis of MM.

**Electronic supplementary material:**

The online version of this article (doi:10.1186/s13046-015-0219-5) contains supplementary material, which is available to authorized users.

## Background

Multiple myeloma (MM) is an incurable cancer of plasma cells caused by abnormal accumulation of monoclonal plasma cells in bone marrow [1]. MM is one of the most common blood cancers and is characterized by wide clinical and pathophysiologic heterogeneities leading to fatal outcome. The survival periods of patients with MM varied significantly, ranging from a few weeks to more than 10 years, and the five-year survival rate is nearly 40 % [2]. Identifying patients who are at the high risk may help optimize the choice of personalized treatment and improve clinical outcomes.

It is well known that the vast majority (>90 %) of the human genome sequence can be actively transcribed, while less than 2 % of transcripts serve as mRNA to encode protein [3, 4]. A substantial fraction of transcripts is non-coding RNA (ncRNA) with no or limited protein coding capacity, including short ncRNA and long ncRNA. Long non-coding RNA (lncRNA), constituting an important class of ncRNA, are mRNA-like transcripts which are transcribed by RNA polymerase II and are longer than 200 nucleotides in length [5, 6]. Accumulating evidence indicates that lncRNA function as important regulators involved in diverse aspects of gene regulation at transcriptional, posttranscriptional and epigenetic levels [5, 7], and participate in a variety of biological processes [8, 9]. The aberrant lncRNA expression has also been observed in many complex human diseases, especially in cancers [10–12]. Similar to mRNA and miRNA, these dysregulated lncRNAs can play oncogenic and/or tumor suppressive roles in the development and progression of cancer. Some well-characterized lncRNAs, such as *MALAT1*, *HOTAIR* and *SRA*, were found to be highly up-regulated in lung cancer, breast cancer, hepatocellular cancer and so on [13–15], while *MEG3*, *GAS5* and *LincRNA-p21* have been shown to be tumor suppressors [16–18]. These cancer-associated lncRNAs displayed aberrant expression patterns in tissue- or cancer-type specific manner [19, 20], suggesting their potentials as novel independent biomarkers for cancer diagnosis and prognosis. Several expression-based lncRNA signatures have been established in glioblastoma multiforme [21], breast cancer [22], oesophageal squamous cell carcinoma [23], colorectal cancer [24] and lung cancer [25]. For multiple myeloma, recent studies have also found that lncRNAs *MALAT1* and *MEG3* are overexpressed in patients with MM compared to healthy individuals by real-time quantitative reverse transcription polymerase chain reactions (RT-PCR) analysis [26, 27]. However, the prognostic significance of lncRNA signature in patients with MM remains unknown.

In the present study, by integrating lncRNA expression profiles and matched clinical information in a large cohort of patients with MM, we identified four prognostic lncRNA biomarkers associated with overall survival of patients with MM and established a four-lncRNA-focus prognostic risk model that can effectively predict clinical survival. The significant prognostic power of four-lncRNA-focus prognostic risk model was further validated in testing dataset and another two independent external patient datasets.

## Methods

### GEO datasets and clinical information of patients with MM

The gene microarray expression data and corresponding clinical information of a large number of patients with MM used in this study were obtained from publicly available Gene Expression Omnibus (GEO) database, including 559 patients from GSE24080 (Affymetrix HG-U133_Plus_2.0 array) (www.ncbi.nlm.nih.gov/geo/query/acc.cgi?acc=GSE24080) [28], 55 patients from GSE57317 (Affymetrix HG-U133_Plus_2.0 array) (www.ncbi.nlm.nih.gov/geo/query/acc.cgi?acc=GSE57317) [29] and 264 patients from GSE9782 (Affymetrix HG-U133A array) (www.ncbi.nlm.nih.gov/geo/query/acc.cgi?acc=GSE9782) [30]. Detailed clinical information of patients with MM used in this study was shown in Additional file 1.

### Microarray analysis and lncRNA re-annotation

The probe ID-centric gene expression data was normalized using the MAS5 algorithm and log2 transformed. To obtain lncRNA expression profiles of patients with MM, the microarray probes were re-annotated as previously described [31]. Briefly, the probes (probe sets) from Affymetrix HG-U133_Plus_2.0 array and Affymetrix HG-U133A array were re-mapped to the human genome (GRCh38) using SeqMap tool [32]. Then those probes (probe sets) that were uniquely mapped to the human genome with no mismatch were retained for further analysis. By matching the chromosomal position of retaining probes (probe sets) to the chromosomal position of lncRNA from the GENCODE project (http://www.gencodegenes.org, release 22) [33], we obtained 3215 probes (probe sets) covering 2330 lncRNAs for Affymetrix HG-U133_Plus_2.0 array and 855 probes (probe sets) covering 663 lncRNAs for Affymetrix HG-U133A array, respectively. The expression data of multiple probes (probe sets) mapping to the same lncRNA were integrated by using the arithmetic mean to represent the expression level of single lncRNA.

### Identification of potential prognostic lncRNA biomarkers associated with OS in patients with MM

A univariate Cox regression analysis was carried out to evaluate the association between expression levels of lncRNAs and patients’ OS. Those lncRNAs whose expression levels were significantly associated with patients’ OS were fitted in a multivariate Cox regression analysis in the training dataset by using OS as the dependent variable and other clinical information as the covariables. We kept those lncRNAs with *p* value <0.01 to develop a risk score model for predicting OS in patients with MM. The lncRNA-based risk score model was defined as the linear combination of the expression values of the prognostic lncRNAs and the multivariable Cox regression coefficient as the weight. The patients with MM in each dataset were classified into high-risk group and low-risk group according to the median risk score derived from the training dataset.

### Statistical analysis

Differences in patients’ OS between high-risk group and low-risk group were demonstrated using the Kaplan-Meier survival curves, and the statistical significance was obtained using the two-sided log-rank test. Univariate and multivariate analyses with Cox proportional hazards regression were carried out with OS as the dependent variable and lncRNA risk score and other individual clinical features as explanatory variables in each dataset. Hazard ratios (HR) and 95 % confidence intervals (CI) were calculated. The time-dependent receiver operating characteristic (ROC) curve was used to evaluate the prognostic performance for survival prediction of the lncRNA risk score and the area under the ROC curves (AUC) value were calculated. All the analysis was performed using the R/Bio-Conductor (version 3.1.1).

### Functional enrichment analysis

The Pearson correlation coefficient was utilized to evaluate co-expression relationship between lncRNA and mRNA. The functional enrichment analysis of co-expressed mRNAs was performed to predict biological function of lncRNA using the DAVID Bioinformatics Tool (http://david.abcc.ncifcrf.gov/, version 6.7), which is widely used bioinformatics resources [34, 35]. The enriched results was reported limited to Gene Ontology (GO) terms in the “Biological Process”(GOTERM-BP-FAT) and Kyoto encyclopedia of genes and genomes (KEGG) pathway categories using the functional annotation clustering and functional annotation chart options. The GO terms and KEGG pathways with *p* value of <0.05 and Enrichment score > 2 was considered as significantly enriched function annotations.

## Results

### Identification of prognostic lncRNA biomarkers associated with patients’ OS from the training dataset

**Fig. 1:**
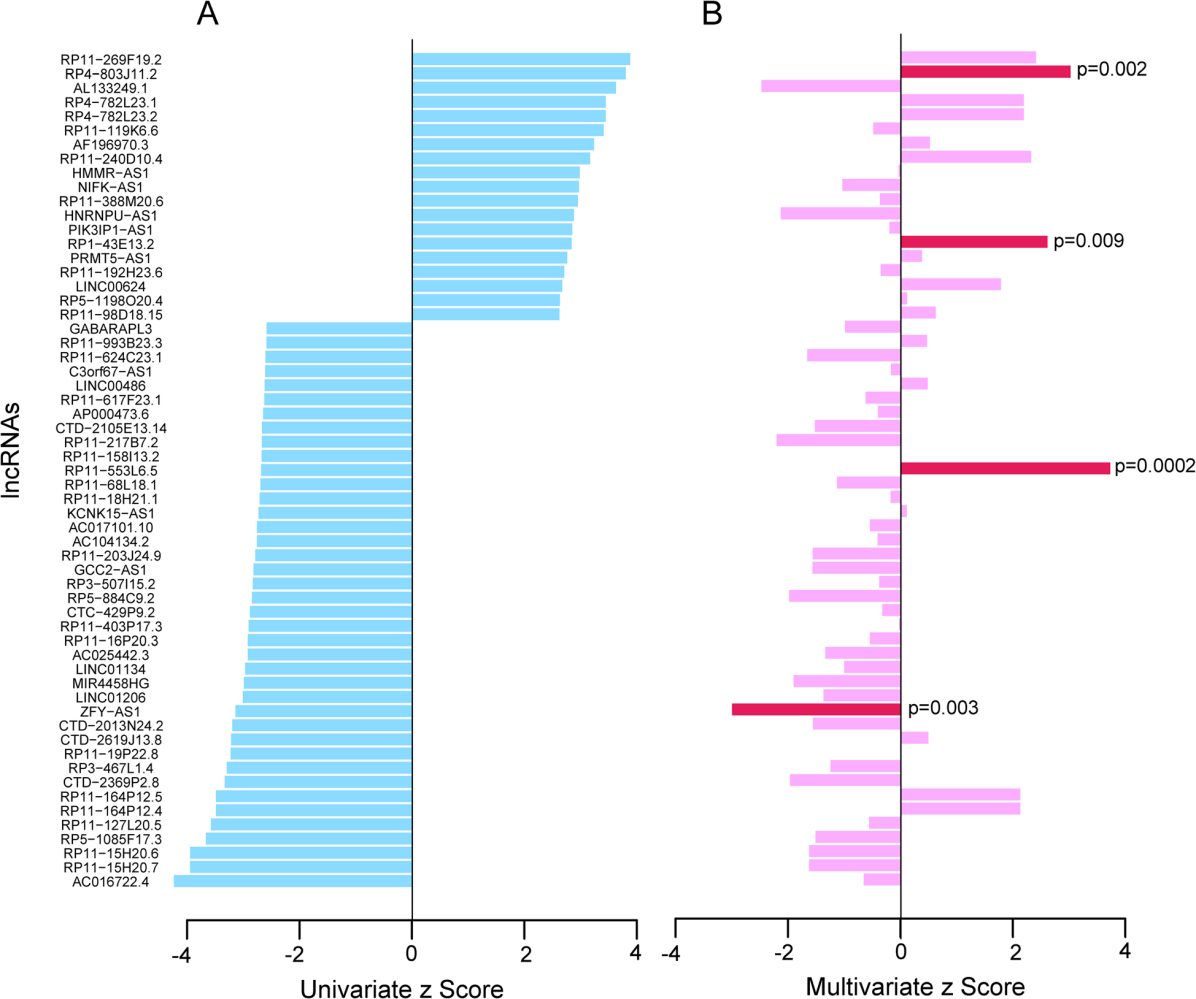
Univariate and multivariate analysis of expression levels of 59 lncRNAs with overall survival as dependent variable. **a** Univariate analyses with Cox proportional hazards regression was carried out to evaluate the association between lncRNA expression and patients’ OS. A total of 59 lncRNAs, whose expression levels were significantly associated with patients’ OS (*p* < 0.01), were identified and ranked according to their univariate z scores. **b** Multivariate analyses with Cox proportional hazards regression was performed on the expression levels of 59 lncRNAs with OS as a dependent variable and other individual clinical features as explanatory variables in the training dataset

The 559 patients with MM from GSE24080 were randomly split into the training dataset (*n* = 280) and the testing dataset (*n* = 279). We first conducted a univariate Cox regression analysis for expression data of each lncRNA with OS as a dependent variable to measure the relationship between lncRNA expression and patients’ OS. A total of 59 lncRNAs, whose expression levels were significantly associated with patients’ OS (*p* < 0.01), were identified and ranked according to their univariate z scores (Fig. 1). Of these, the high expression levels of 40 lncRNAs with negative z scores were associated with longer OS, and high expression levels of the remaining 19 lncRNAs with positive z scores were associated with shorter OS. In order to evaluate whether these lncRNAs have independently predictive power to predict patients’ OS when considering the mutual effect among 59 lncRNAs and clinical features, a multivariate regression analysis was further performed on the expression levels of 59 lncRNAs with OS as a dependent variable and other individual clinical features as explanatory variables in the training dataset. When considering the mutual effect among 59 lncRNAs and clinical features, only 4 of 59 lncRNAs (*RP4-803 J11.2*, *RP1-43E13.2*, *RP11-553 L6.5*, *ZFY-AS1*) showed predictive power and were able to independently predict patients’ OS at a statistically significant level of 0.01 (Fig. 1). The detailed information of these four lncRNAs was summarized in Table 1. To build a predictive model that should be independent of other factors (such as clinical features or other lncRNAs), we only used these four lncRNAs to construct risk score model.

**Table 1:** The detailed information of four prognostic lncRNAs for OS in patients with MM

Ensembl id	Gene symbol	Chromosomal position	*P* value^a^	Hazard ratio^a^	Coefficient^a^
ENSG00000237481	*RP4-803 J11.2*	Chromosome 1: 229,319,403–229,323,087(+)	1.42E-04	1.429	0.357
ENSG00000230424	*RP1-43E13.2*	Chromosome 1: 19,210,501–19,240,704(+)	0.005	1.656	0.504
ENSG00000259976	*RP11-553 L6.5*	Chromosome 3: 114,314,501–114,316,179(−)	0.007	0.702	−0.354
ENSG00000233070	*ZFY-AS1*	Chromosome Y: 2,966,844–3,002,626(−)	0.002	0.758	−0.276

^a^Derived from the univariate Cox regression analysis in the 280 patients of training dataset

### Construction and validation of lncRNA-focus risk score model for predicting OS in the training dataset

**Fig. 2:**
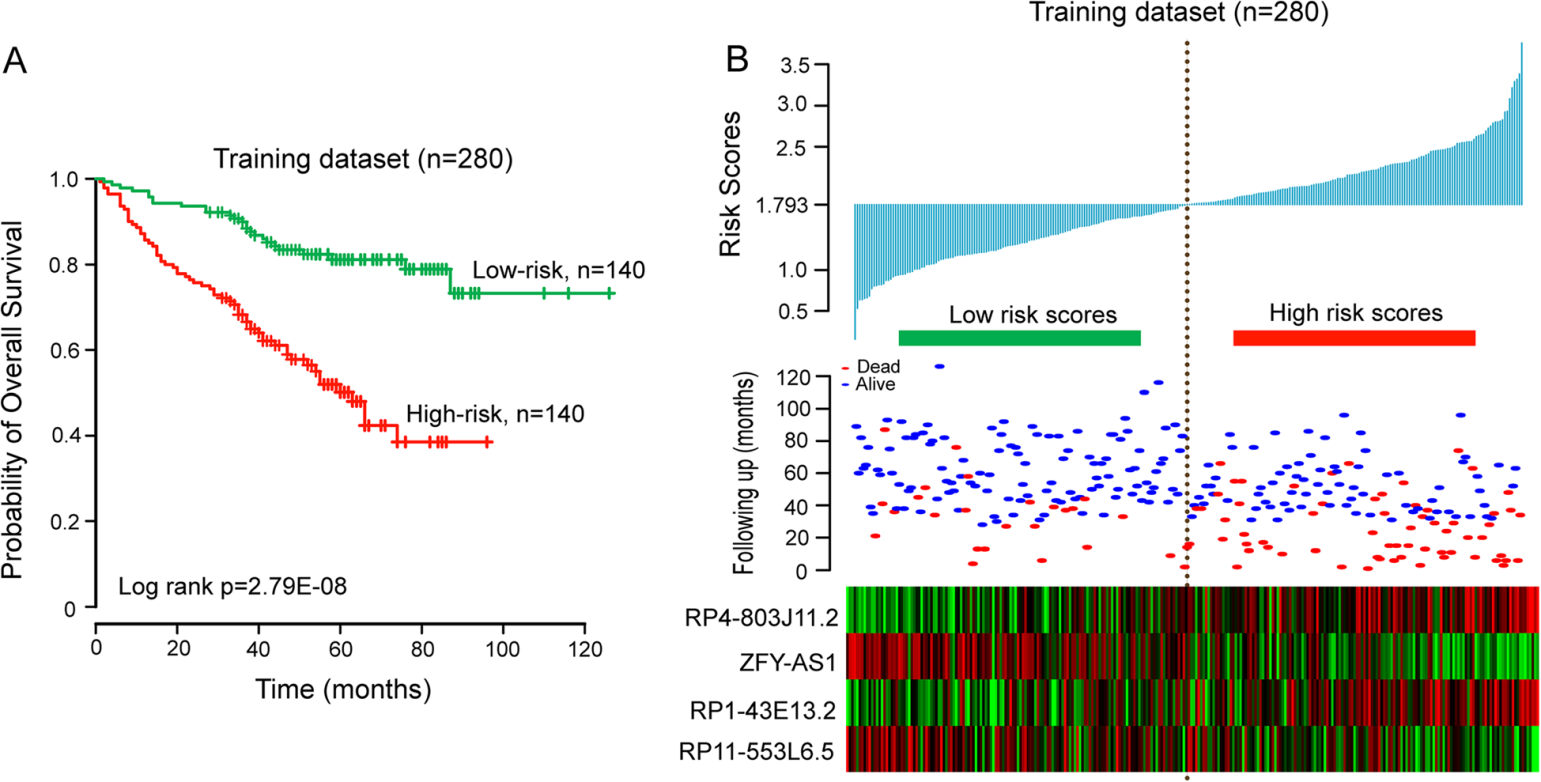
The four-lncRNA-focus risk score model predicts overall survival of patients with MM in the training dataset. **a** Kaplan-Meier analysis for overall survival of patient with high-risk or low-risk scores. *P* value was calculated using the two-sided log-rank test. **b** Expression pattern of four prognostic lncRNAs that correlates with patients’ survival status and increased risk scores

To construct a predictive model, these four lncRNAs were fitted in a multivariate Cox regression model with OS as a dependent variable to measure relative contributions for survival prediction. Then a lncRNA-focus risk score model for OS prediction was developed by integrating the expression data of these four lncRNA and corresponding coefficient derived from above multivariate regression analysis, as follows: Risk score = (0.3016 × expression value of *RP4-803 J11.2*) + (−0.2989 × expression value of *ZFY-AS1*) + (0.3191 × expression value of *RP1-43E13.2*) + (−0.1445 × expression value of *RP11-553 L6.5*). The risk score of each patient in the training dataset was calculated according to the lncRNA-focus risk score model. Then 280 patients of training dataset were assigned to a high-risk group (*n* = 140) or a low-risk group (*n* = 140) using the median risk score as the cutoff point. The result of Kaplan-Meier analysis showed significant differences in patients’ OS between high-risk group and low-risk group (log-rank test *p* < 0.001, Fig. 2a). Patients in the high-risk group had significantly shorter OS (mean 58.7 months) than those in the low-risk group (mean 104.6 months). The univariate Cox regression analysis also demonstrated that the risk scores derived from the four-lncRNA signature was significantly correlated with patients’ OS with risk scores as a continuous variable (*p* < 0.001, HR = 2.718, 95 % CI = 1.937–3.815) (Table 2). The expression of lncRNAs *RP4-803 J11.2* and *RP1-43E13.2* tended to be up-regulated and the remaining two lncRNAs (*ZFY-AS1* and *RP11-553 L6.5*) were down-regulated for patients in high-risk group (Fig. 2b).

**Table 2:** Univariable and multivariable Cox regression analysis of the four-lncRNA signature and overall survival in each dataset

Variables	Univariate analysis^a^			Multivariable analysis^a^		
	HR	95 % CI of HR	*P* Value	HR	95 % CI of HR	*P* Value
Training dataset (*n* = 280)						
lncRNA-focus risk score	2.718	1.937–3.815	7.262E-09	2.066	1.395–3.060	2.94E-04
Age	1.032	1.008–1.056	0.008	1.020	0.997–1.044	0.090
Gender (female/male)	0.844	0.556–1.280	0.424	1.047	0.665–1.648	0.842
Total Therapy (TT2/TT3)	0.914	0.572–1.462	0.709	1.060	0.648–1.735	0.816
IgA isotype (N/Y)	1.044	0.654–1.666	0.857	1.032	0.794–2.134	0.295
Serum beta 2-microglobulin ≥ 3.5 mg/L (N/Y)	2.677	1.742–4.112	6.99E-06	1.733	1.057–2.843	0.029
C-reactive protein ≥ 8.0 mg/L (N/Y)	1.749	1.155–2.648	0.008	1.008	0.628–1.616	0.975
Creatinine ≥ 2.0 mg/dL (177 μmol/L) (N/Y)	3.821	2.338–6.245	8.84E-08	1.864	0.992–3.501	0.053
Lactate dehydrogenase > upper limit of normal (>190 U/L) (N/Y)	2.651	1.749–4.017	4.33E-06	1.484	0.928–2.371	0.099
Serum albumin <35 g/ L (N/Y)	2.003	1.228–3.266	0.005	1.559	0.929–2.618	0.093
Testing dataset (*n* = 279)						
lncRNA-focus risk score	1.579	1.099–2.270	0.014	1.726	1.113–2.675	0.015
Age	1.015	0.991–1.039	0.223	1.006	0.982–1.031	0.616
Gender (female/male)	1.130	0.722–1.768	0.593	1.667	0.981–2.831	0.059
Total Therapy (TT2/TT3)	0.651	0.368–1.150	0.139	0.590	0.329–1.060	0.077
IgA isotype (N/Y)	1.189	0.724–1.953	0.494	1.428	0.837–2.438	0.192
Serum beta 2-microglobulin ≥ 3.5 mg/L (N/Y)	1.866	1.206–2.888	0.005	1.470	0.886–2.437	0.136
C-reactive protein ≥ 8.0 mg/L (N/Y)	1.233	0.786–1.932	0.362	1.158	0.734–1.828	0.529
Creatinine ≥ 2.0 mg/dL (177 μmol/L) (N/Y)	1.774	0.937–3.359	0.078	0.954	0.467–1.946	0.896
Lactate dehydrogenase > upper limit of normal (>190 U/L) (N/Y)	1.993	1.283–3.097	0.002	1.918	1.133–3.249	0.015
Serum albumin <35 g/ L (N/Y)	1.877	1.055–3.340	0.032	1.791	0.933–3.437	0.080
Entire GSE24080 dataset (*n* = 559)						
lncRNA-focus risk score	2.099	1.638–2.688	4.404E-09	1.905	1.434–2.530	8.65E-06
Age	1.024	1.007–1.041	0.005	1.013	0.996–1.030	0.128
Gender (female/male)	0.973	0.717–1.319	0.860	1.338	0.955–1.875	0.090
Total Therapy (TT2/TT3)	0.797	0.556–1.143	0.218	0.805	0.554–1.168	0.254
IgA isotype (N/Y)	1.106	0.787–1.555	0.561	1.261	0.888–1.791	0.194
Serum beta 2-microglobulin ≥ 3.5 mg/L (N/Y)	2.236	1.651–3.028	2.0E-07	1.574	1.111–2.231	0.011
C-reactive protein ≥ 8.0 mg/L (N/Y)	1.485	1.097–2.011	0.011	1.123	0.818–1.543	0.474
Creatinine ≥ 2.0 mg/dL (177 μmol/L) (N/Y)	2.730	1.856–4.015	3.35E-07	1.377	0.877–2.160	0.165
Lactate dehydrogenase > upper limit of normal (>190 U/L) (N/Y)	2.317	1.714–3.133	4.77E-08	1.645	1.180–2.294	0.003
Serum albumin <35 g/ L (N/Y)	1.946	1.342–2.821	4.47E-04	1.519	1.028–2.245	0.036
GSE57317 dataset (*n* = 55)^b^						
lncRNA-focus risk score	2.640	1.013–6.879	0.047			
GSE9782 dataset (*n* = 264)						
lncRNA-focus risk score	1.637	1.107–2.42	0.014	1.909	1.269–2.870	0.002
Age	1.014	0.998–1.03	0.087	1.016	0.999–1.032	0.054
Gender (female/male)	1.334	0.961–1.853	0.086	1.543	1.098–2.169	0.012

^a^lncRNA-focus risk score and age were evaluated as continuous variables in both univariate and multivariate Cox regression analysis

^b^There was no available clinical features in GSE57317 dataset

**Table 3:** Top six enriched functional clusters of GO terms and KEGG pathways

GO terms and KEGG pathways	NO. of genes	*P*-value	Fold enrichment
Cluster 1 (Enrichment Score: 11.01)			
GO:0000280 ~ nuclear division	33	4.55E-14	5.14
GO:0007067 ~ mitosis	33	4.55E-14	5.14
GO:0000279 ~ M phase	40	6.73E-14	4.16
GO:0000087 ~ M phase of mitotic cell cycle	33	7.61E-14	5.05
GO:0048285 ~ organelle fission	33	1.42E-13	4.94
GO:0022403 ~ cell cycle phase	43	1.43E-12	3.56
GO:0007049 ~ cell cycle	61	2.78E-12	2.69
GO:0000278 ~ mitotic cell cycle	39	1.25E-11	3.61
GO:0022402 ~ cell cycle process	47	2.41E-10	2.85
GO:0051301 ~ cell division	31	2.50E-09	3.60
GO:0000070 ~ mitotic sister chromatid segregation	12	3.47E-09	11.42
GO:0000819 ~ sister chromatid segregation	12	4.82E-09	11.11
GO:0007059 ~ chromosome segregation	16	9.96E-09	6.77
Cluster 2 (Enrichment Score: 4.27)			
GO:0051276 ~ chromosome organization	38	8.45E-08	2.68
GO:0016568 ~ chromatin modification	19	0.001137	2.37
GO:0006325 ~ chromatin organization	23	0.001656	2.08
Cluster 3 (Enrichment Score: 3.58)			
GO:0006260 ~ DNA replication	20	2.88E-06	3.61
hsa03030:DNA replication	7	9.85E-04	5.89
GO:0006261 ~ DNA-dependent DNA replication	7	0.006632	4.13
Cluster 4 (Enrichment Score: 3.33)			
GO:0007051 ~ spindle organization	8	2.94E-04	6.09
GO:0000226 ~ microtubule cytoskeleton organization	14	3.64E-04	3.26
GO:0007017 ~ microtubule-based process	19	4.48E-04	2.57
GO:0007010 ~ cytoskeleton organization	26	0.001002	2.04
Cluster 5 (Enrichment Score: 2.59)			
GO:0006259 ~ DNA metabolic process	34	1.31E-05	2.30
GO:0006974 ~ response to DNA damage stimulus	21	0.006553	1.93
GO:0006281 ~ DNA repair	16	0.019452	1.93
GO:0033554 ~ cellular response to stress	26	0.025459	1.57
Cluster 6 (Enrichment Score: 2.57)			
GO:0008380 ~ RNA splicing	23	3.03E-05	2.77
GO:0006397 ~ mRNA processing	23	1.82E-04	2.454
GO:0016071 ~ mRNA metabolic process	24	5.39E-04	2.22
GO:0006396 ~ RNA processing	31	6.70E-04	1.94
GO:0000398 ~ nuclear mRNA splicing, via spliceosome	11	0.014111	2.46
GO:0000377 ~ RNA splicing, via transesterification reactions with bulged adenosine as nucleophile	11	0.014111	2.46
GO:0000375 ~ RNA splicing, via transesterification reactions	11	0.014111	2.46
hsa03040:Spliceosome	10	0.022065	2.40

### Performance evaluation of lncRNA-focus risk score model for survival prediction in the testing and entire GSE24080 datasets

**Fig. 3:**
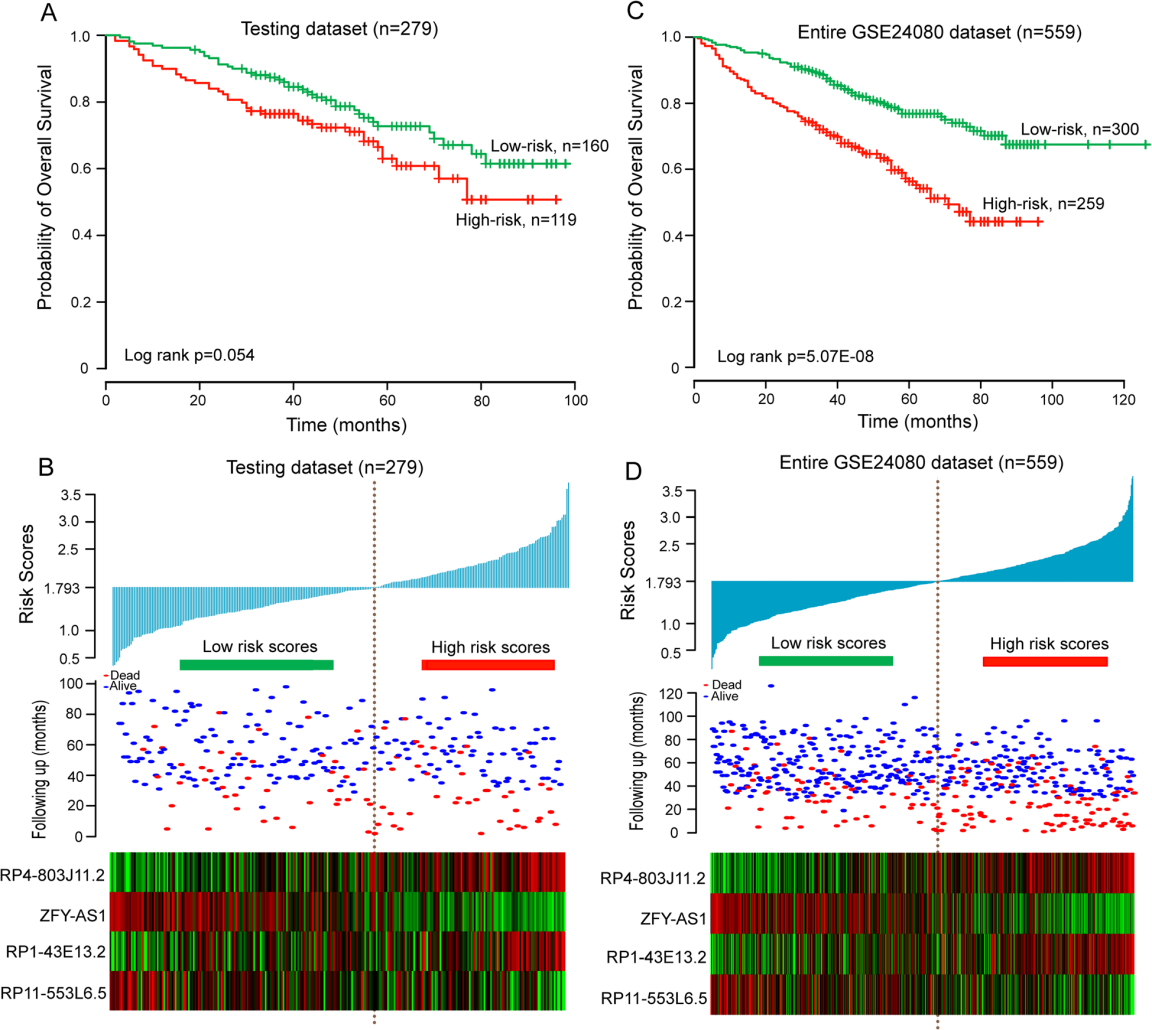
The four-lncRNA-focus risk score model predicts overall survival of patients with MM in the testing and entire GSE24080 datasets. **a** Kaplan-Meier analysis for overall survival of patient with high-risk or low-risk scores in the testing dataset. **b** The risk score distribution, survival status and expression pattern of four prognostic lncRNAs in 279 patients of testing dataset. **c** Kaplan-Meier analysis for overall survival of patient with high-risk or low-risk scores in entire GSE24080 dataset. **d** The risk score distribution, survival status and expression pattern of four prognostic lncRNAs in 559 patients of GSE24080 dataset

To evaluate the prognostic power of lncRNA-focus risk score model for patients’ OS prediction, this risk score model and cutoff point derived from the training dataset was applied to patients with MM in the testing dataset and the entire GSE24080 dataset. The 279 patients of the testing dataset were classified into either high-risk group (*n* = 119) or low-risk group (*n* = 160). Kaplan-Meier curves for the two groups within the testing dataset is shown in Fig. 3a, demonstrating a significant difference in OS between high-risk group and low-risk group (log-rank test *p* = 0.054). Patients in the high-risk group exhibited poorer OS (mean 68.5 months) than those in the low-risk group (mean 78.3 months). The significant association between risk score and OS has also been observed in the testing dataset with risk scores as a continuous variable in the univariate Cox regression analysis (*p* = 0.014, HR = 1.579, 95 % CI = 1.099–2.270) (Table 2). The distribution of risk score, survival status and lncRNA expression in the testing dataset of 279 patients is shown in Fig. 3b. Patients in the high-risk group tended to express risky lncRNAs (*RP4-803 J11.2* and *RP1-43E13.2*) at higher level than those in the low-risk group, whereas patients in the low-risk group tended to express protective lncRNAs (*ZFY-AS1* and *RP11-553 L6.5*) at higher level than those in the high-risk group. In consistent with the finding in the training dataset and testing dataset, Kaplan-Meier and univariate Cox regression analysis showed that this lncRNA-focus risk score model was able to separate 559 patients in the entire GSE24080 dataset into two groups with significantly different OS (mean 63.3 months versus 100.1 months, HR = 2.099, 95 % CI = 1.638–2.688; *p* < 0.001, log-rank test) (Fig. 3c). The distribution of risk score, survival status and lncRNA expression also yielded similar results (Fig. 3d).

### Further validation of lncRNA-focus risk score model for survival prediction in another two independent external patient datasets

**Fig. 4:**
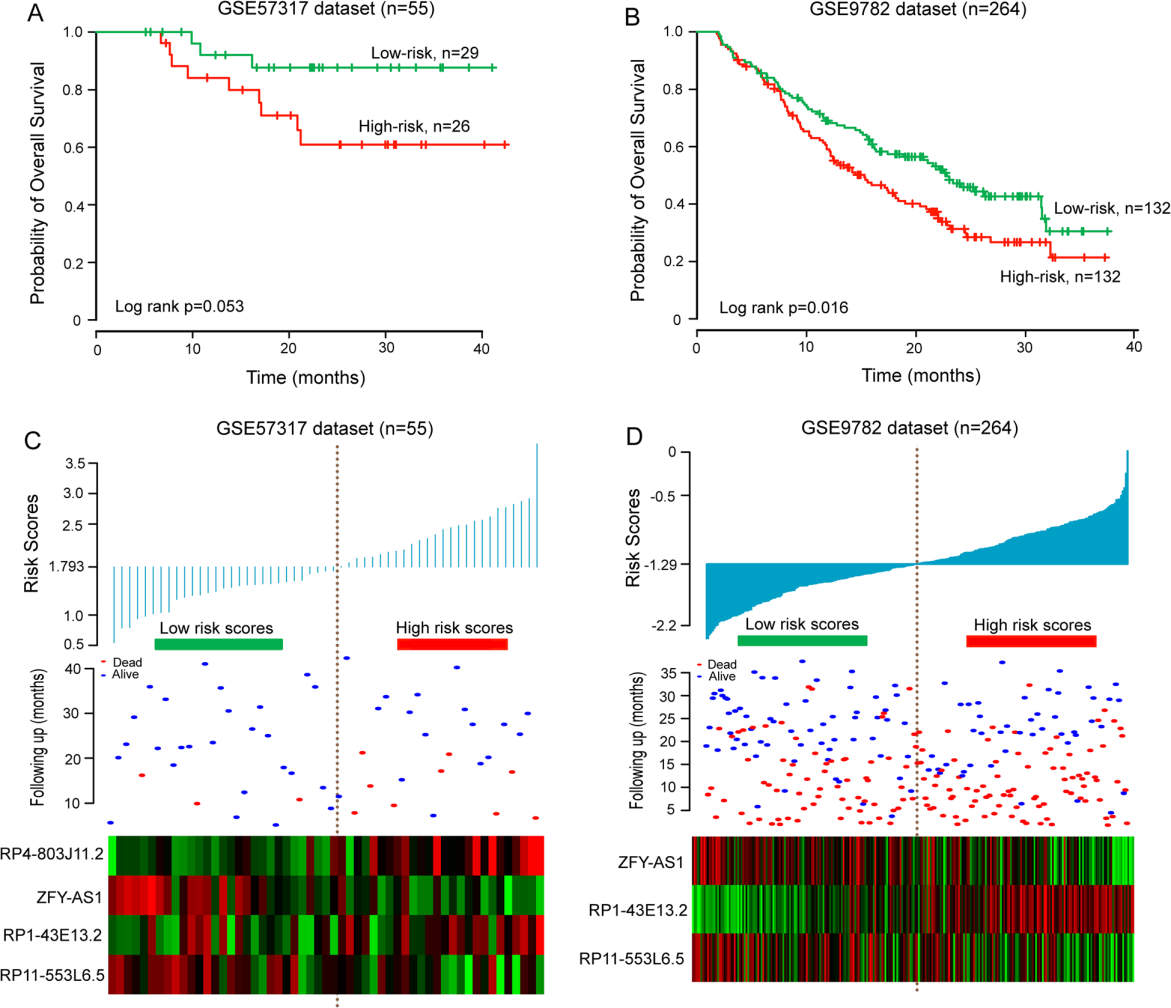
Performance validation of lncRNA-focus risk score model for survival prediction in another two independent external patient datasets. **a** Kaplan-Meier estimates for overall survival of patients in the GSE57317 dataset. **b** Kaplan-Meier estimates for overall survival of patients in the GSE9782 dataset. **c** The risk score distribution, survival status and expression pattern of four prognostic lncRNAs in 55 patients of GSE57317 dataset. **d** The risk score distribution, survival status and expression pattern of three prognostic lncRNAs in 264 patients of GSE9782 dataset

To further examine the robustness and practical application of the four-lncRNA risk score model, we validated the prognostic power of the risk score model using lncRNA expression values and survival information of patients with MM in another two independent external datasets (GSE57317 and GSE9782). As shown in Fig. 4a, the lncRNA-focus risk score model could effectively predict OS in patients with MM from GSE57317 (log-rank test *p* = 0.053). All 55 patients in the GSE57317 dataset were divided into the high-risk group (*n* = 26) and the low-risk group (*n* = 29) with significant different OS according to the same risk score cutoff point derived from the training dataset (mean 31.3 months versus 37.5 months, HR = 2.64, 95 % CI = 1.013–6.879, *p* = 0.047). Another external patient dataset (GSE9782) was based on the Affymetrix U133A array platform. After probe re-annotating, we found that only 3 lncRNA (*RP1-43E13.2*, *RP11-553 L6.5*, *ZFY-AS1*) of four prognostic lncRNAs from the training dataset were covered on the Affymetrix U133A array. So, the risk score model only based on these three lncRNAs without re-estimating parameters was used to predict OS for GSE9782 dataset, which perhaps reduce the predictive power. The median risk score cutoff point obtained from GSE9782 dataset classified 264 patients into the high-risk group (*n* = 132) and the low-risk group (*n* = 132). The Kaplan-Meier curves for the high-risk group and the low-risk group in the independent external GSE9782 dataset are shown in Fig. 4b. Patients assigned into high-risk group tended to have shorter OS than those in the low-risk group (mean OS 18.4 months vs. 22.4 months, log-rank test *p* = 0.016). The univariate Cox regression analysis also showed that the risk scores were significantly associated with OS in patients with MM in the GSE9782 dataset (HR = 1.637, 95 % CI = 1.107–2.42, *p* = 0.014). The results of risk score distribution, survival status and lncRNA expression for GSE57317 and GSE9782 were consistent with those observed in the training dataset (Fig. 4c and d).

### Independence of lncRNA-focus risk score model for survival prediction from clinical features

**Fig. 5:**
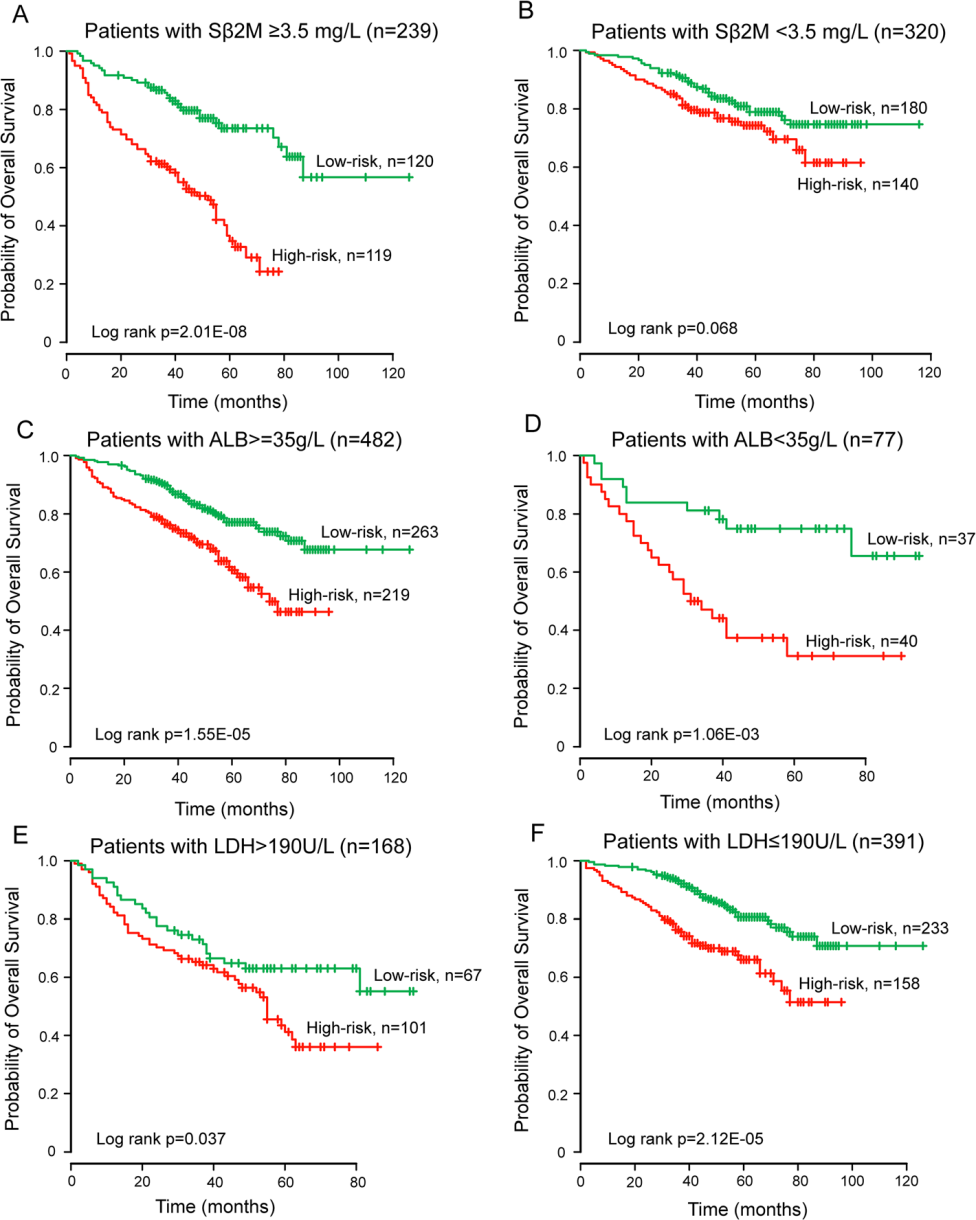
Survival analysis of all patients with available Sβ2M, ALB and LDH information. **a** Kaplan-Meier curves for patients with higher Sβ2M level (≥3.5 mg/L). **b** Kaplan-Meier curves for patients with lower Sβ2M level (<3.5 mg/L). **c** Kaplan-Meier curves for patients with higher ALB (≥35 g/L). **d** Kaplan-Meier curves for patients with lower ALB (<35 g/L). **e** Kaplan-Meier curves for patients with higher LDH (>190 U/L). **f** Kaplan-Meier curves for patients with lower LDH (≤190 U/L)

To assess whether the prognostic values of lncRNA-focus risk score model is independent of other important clinical features of patients with MM, the multivariate Cox regression analyses were performed with OS as the dependent variable and lncRNA risk score and other clinical features as explanatory variables in each dataset. The multivariate Cox regression analyses showed that lncRNA-focus risk score was significantly correlated with OS of patients with MM after adjusting for various clinical features in the training dataset (HR = 2.066, CI = 1.395–3.06, *p* < 0.001), testing dataset (HR = 1.726, CI = 1.113–2.675, *p* = 0.015), GSE24080 dataset (HR = 1.905, CI = 1.434–2.53, *p* < 0.001) and another independent external patient dataset GSE9782 (HR = 1.909, CI = 1.269–2.87, *P* = 0.002; Table 2) (There was no available clinical features in GSE57317 dataset). We also found that higher levels of serum beta 2-microglobulin (Sβ2M), serum albumin (ALB) and lactate dehydrogenase (LDH) were significant in the multivariate analysis. However, the estimation of hazard ratios of lncRNA-focus risk score for OS is 1.905 (*p* < 0.001) is higher than that of Sβ2M (HR = 1.574, *p* = 0.011), ALB (HR = 1.519, *p* = 0.036) and LDH (HR = 1.645, *p* = 0.003) (Table 2), suggesting that lncRNA-focus risk score model may be more powerful prognostic factor than established laboratory prognostic parameters. Next, data stratification analysis was then performed according to these three significant clinical features. All patients of GSE24080 were stratified into patient group with higher Sβ2M level (≥3.5 mg/L) or patient group with lower Sβ2M level (<3.5 mg/L). All 239 patients with higher Sβ2M level were divided into the high-risk group (*n* = 119) with shorter OS or the low-risk group (*n* = 120) with longer OS (mean 44.9 vs. 93.4 months, log-rank test *p* < 0.001) (Fig. 5a). For the patient with lower Sβ2M level, patients with low-risk scores (*n* = 180) also had longer OS (mean 97.1 months) than those with high-risk scores (*n* = 140) (mean 75.2 months), although the *p* value of 0.068 was slightly above the 0.05 significance level (Fig. 5b). Another clinical feature, ALB, stratified the entire GSE24080 patients into two subgroups with higher (≥35 g/L) or lower (<35 g/L) levels of ALB. The lncRNA-focus risk score model could effectively classify patients into high-risk group and low-risk group with significantly different OS for both two subgroups with higher or lower levels of ALB (mean 66.4 vs. 100.8 months, log-rank test *p* < 0.001 for 482 patients with higher level of ALB, and mean 44 vs. 74.1 months, log-rank test *p* = 0.001 for 77 patients with lower level of ALB) (Fig. 5c and d). Significant differences for OS between high-risk group and low-risk group also were observed for stratified subgroup by LDH level (mean 51.5 vs. 67.9 months, log-rank test *p* = 0.037 for 168 patients with LDH > 190 U/L, and mean 69.1 vs. 104.4 months, log-rank test *p* < 0.001 for 391 patients with LDH ≤ 190 U/L) (Fig. 5e and f). Taken together, the results of multivariate Cox regression analyses and stratification analysis suggested that the predictive power of lncRNA-focus risk score is independent of other clinical features for OS of patients with MM.

### Performance comparison by time-dependent ROC curve analysis

**Fig. 6:**
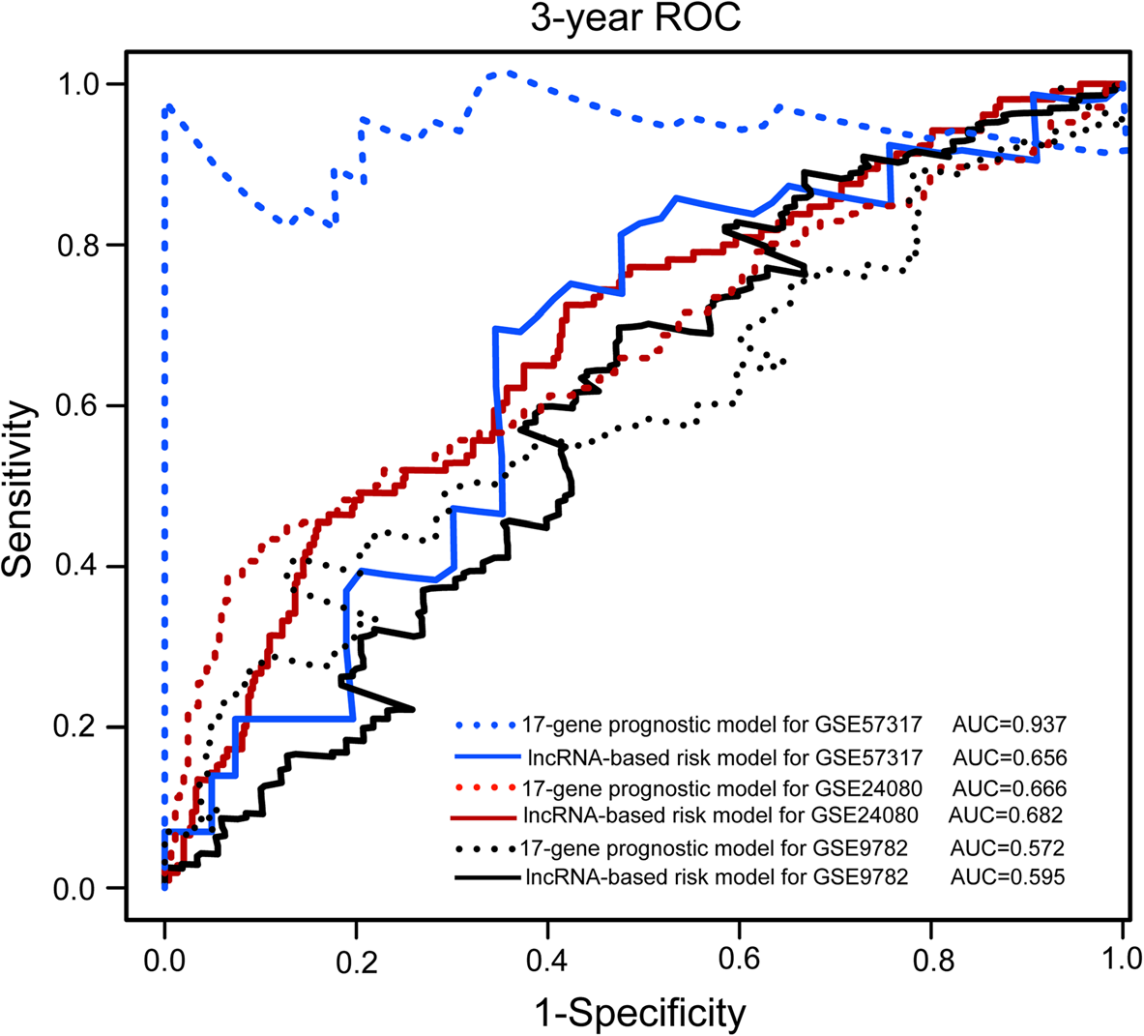
ROC analysis of the sensitivity and specificity for survival prediction by lncRNA-based risk model and 17-gene prognostic model. The time-dependent ROC curve was used to evaluate the prognostic performance for survival prediction. Performance comparison was assessed between four-lncRNA signature and 17-gene signature by calculating the area under the ROC curves (AUC) in three datasets

We performed the time-dependent ROC curve analysis to compare sensitivity and specificity for survival prediction between lncRNA-focus risk score model and an established UAMS 17-gene prognostic model [36] in the GSE24080 dataset, GSE57317 dataset and GSE9782. The AUC value was obtained from ROC analysis and compared between these two predictive models. In the GSE24080 dataset and GSE9782 dataset, the lncRNA-focus risk score model achieved AUC value of 0.682 and 0.595, which is higher than those (AUC = 0.666 and 0.572) derived from UAMS 17-gene prognostic model (Fig. 6), indicating that the predictive ability of lncRNA-focus risk score model was better than UAMS 17-gene prognostic model in GSE24080 and GSE9782 datasets. However, in the GSE57317 dataset, established UAMS 17-gene prognostic model had a higher AUC value than our lncRNA-focus risk score model (0.937 vs. 0.656, Fig. 6).

### Functional prediction of prognostic lncRNA biomarkers

To explore the functional implication of four prognostic lncRNA biomarkers in MM tumorigenesis and development, we performed bioinformatics analysis to predict lncRNA functions. We first calculated the Pearson correlation coefficient between lncRNA and mRNA by examining the paired lncRNA and mRNA expression profiles of 559 patients with MM in the GSE24080 dataset. The top 1 % mRNA was selected as co-expressed mRNAs with prognostic lncRNA biomarkers. A total of 789 mRNAs were positively or negatively correlated with at least one of the four prognostic lncRNAs (see Additional file 2). Functional enrichment analysis showed that these co-expressed mRNAs with prognostic lncRNAs were significantly enriched in 104 GO terms and 9 KEGG pathways (*p* < 0.05 and Fold Enrichment > 2) (see Additional file 3), which are mainly involved in six functional clusters including cell cycle, chromatin modification, DNA replication, microtubule-based process, DNA repair and RNA processing (Table 3). We further examined whether there were any important genes of interest identified from integrative analysis of lncRNA-mRNA. We found that 135 of 789 co-expressed mRNAs (corresponding to 62 genes) with four prognostic lncRNAs are known cancer genes recorded in NCG database (http://ncg.kcl.ac.uk/index.php, version 4.0) [37] (see Additional file 4), which is a manually curated cancer gene repository. Especially, gene *NRAS* has been verified experimentally to be associated with MM [38].

## Discussion

During the past years, great progress in our understanding of the initiation and progression of multiple myeloma has been witnessed [39, 40]. However, the clinical outcome of patients with MM still remains highly heterogeneous. Traditional laboratory parameters Sβ2M and serum albumin, referred to the International Staging System (ISS), have been used as an objective staging system [41]. Subsequent cytogenetic studies found that cytogenetic abnormalities, such as 13q14 deletion and t(4;14) translocation, also can provide valuable prognostic information [42, 43]. However, both ISS and cytogenetic abnormalities demonstrated limited ability for therapeutic risk stratification. With the development of high-throughput techniques, expression profiles-based molecular signatures have been reported in various types of cancers and have become more powerful prognostic tool to predict patient outcomes [44, 45]. Several multigene-expression signatures, including UAMS 17-gene [36], IFM 15-gene [2] and EMC 92-gene [46] models, have been developed to predict survival in patients with MM. More recently, dysregulation of lncRNA expression were observed in the newly diagnosed patients with MM, indicating their potentials as biomarkers for diagnosis and prognosis in MM [27]. However, the prognostic significance of expression profile-based lncRNA signature for outcome prediction in patients with MM has not yet been investigated.

In this study, we have investigated the lncRNA expression profiles of a large cohort of patients with MM by repurposing the publically available microarray data. Through integrative analysis of lncRNA expression data with clinical features, 59 lncRNAs were found to be significantly associated with patients’ OS in MM. After considering interrelation among 59 lncRNAs and clinical features using multivariate analysis, we identified four prognostic lncRNAs that were able to independently predict patients’ OS. Two (*RP4-803 J11.2* and *RP1-43E13.2*) of four lncRNAs are located chromosome 1 that has been proven to be key players in MM progression [36], and their expression correlated with shortened survival. Then an lncRNA-focus risk model was developed by incorporating expression patterns of four prognostic lncRNAs and their relative contributions in the multivariate analysis in the training dataset. By applying this four-lncRNA-based risk model to the patients of training dataset, a better risk stratification for patients’ outcome was observed between survival curves of patients with high-risk or low-risk scores. Patients in the high-risk group had significant shorter OS than those in the low-risk group. Further validation of the four-lncRNA-based risk model constructed in the training dataset showed similar prognostic power in the testing dataset and another two independent external patient datasets. These analyses suggested that the prognostic value of the four-lncRNA-based risk model is robust and reliable for survival prediction in MM.

We next performed multivariate analysis to test whether the prognostic power of the four-lncRNA-based risk model for survival prediction is independent of known prognostic variables and other clinical features. The estimations of HR for OS were 2.066, 1.726, 1.905 and 1.909 in the training, testing, entire GSE24080 and GSE9782 datasets, respectively. Also, some known prognostic variables, including Sβ2M, serum albumin and LDH revealed significant correlation with patients’ OS. So we carried out stratification analysis for Sβ2M, ALB and LDH to further evaluate the independence of the four-lncRNA-based risk model for survival prediction. The results of stratification analysis suggested that the four-lncRNA-based risk model was able to effectively classify patients into high-risk group and low-risk group with significantly different OS for both two subgroups stratified by three clinical prognostic variables. These results of multivariate analysis, taken together with stratification analysis, demonstrated that the four-lncRNA-based risk model was an independent prognostic factor for survival prediction in MM.

Although substantial computational evidence for the prognostic significance of the lncRNA signature in MM has been revealed, the underlying mechanisms of these four prognostic lncRNAs in the development of MM were still unclear. So we performed an integrative analysis of lncRNA-mRNA by utilizing the matched lncRNA and mRNA expression profiles to infer functional implication of these four prognostic lncRNAs. The functional enrichment analysis of mRNA co-expressed with lncRNAs revealed that the biological functions annotated to the four prognostic lncRNAs mainly involve cell cycle, chromatin modification, DNA replication, microtubule-based process, DNA repair and RNA processing. These functions are all of essential significance contributing to the initiation and progression of MM [39]. Although bioinformatics analysis indicated that these four prognostic lncRNAs may play significant role in the initiation and progression of MM through associations with known genetic and epigenetic events linked to MM, further experimental validation of these four prognostic lncRNAs is necessary for understanding their functional roles in MM.

## Conclusions

In summary, we identified four prognostic lncRNA biomarkers that are significantly associated with OS of patients with MM and developed an lncRNA-focus risk model for survival prediction by integrating lncRNA expression profiles with clinical features of a large cohort of patients with MM. The four-lncRNA signature could robustly predict OS of patients with MM. The prognostic power of the four-lncRNA signature was independent of known laboratory prognostic factors and other clinical features, and exhibited superior performance compared to known traditional clinical parameters and multigene signature to some extent. These results demonstrated potential application of lncRNAs as novel independent biomarkers for diagnosis and prognosis in MM. Moreover, identification of lncRNA biomarkers perhaps brings novel insights into the understanding of underlying molecular basis of MM.

## Additional files

Additional file 1: Table S1. Clinical and pathological characteristics of patients with MM in our study. (DOC 36 kb) Additional file 2: Table S2. List of mRNAs co-expressed with at least one of the four prognostic lncRNAs. (XLS 131 kb) Additional file 3: Table S3. List of enriched GO terms and KEGG pathways of mRNAs co-expressed with prognostic lncRNAs. (XLS 72 kb) Additional file 4: Table S4. List of known cancer genes recorded in NCG database which were co-expressed with four prognostic lncRNAs. (XLSX 13 kb)

## Abbreviations

ALB: Albumin
AUC: Area under the ROC curve
CI: Confidence interval
CRC: Colorectal cancer
GEO: Gene Expression Omnibus
GO: Gene Ontology
HR: Hazard ratio
ISS: International Staging System
KEGG: Kyoto Encyclopedia of Genes and Genomes
LDH: Lactate dehydrogenase
LncRNAs: Long non-coding RNAs
MM: Multiple myeloma
OS: Overall survival
RT-PCR: Reverse transcription polymerase chain reactions
ROC: Receiver operating characteristic
Sβ2M: Serum beta 2-microglobulin
