# Supplementary material for: Identification and validation of potential prognostic lncRNA biomarkers for predicting survival in patients with multiple myeloma
Source: J Exp Clin Cancer Res. 2015 Sep 11;34(1):102. doi: 10.1186/s13046-015-0219-5 (PMC4567800; doi:10.1186/s13046-015-0219-5)
Supplement: Additional file 1: Table S1. — Clinical and pathological characteristics of patients with MM in our study. (DOC 36 kb) [file 13046_2015_219_MOESM1_ESM.doc]

**Supplementary Table S1.** Clinical and pathological characteristics of patients with MM in our study.

| Characteristics | Training dataset  (n=280) | Testing dataset  (n=279) | Entire GSE24080 dataset  (n=559) | GSE57317 dataset  (n=55) | GSE9782 dataset  (n=264) |
| --- | --- | --- | --- | --- | --- |
| Age≥65y | 66 (23.6) | 70(25.1) | 136(24.3) |  | 87(33.0) |
| Female | 112(40.0) | 110(39.4) | 222(39.7) |  | 105(39.8) |
| IgA isotype | 71(25.4) | 62(22.2) | 133(23.8) |  |  |
| Beta 2-microglobulin≥3.5 mg/L | 126(45.0) | 113(40.5) | 239(42.8) |  |  |
| C-reactive protein≥8.0 mg/L | 99(35.4) | 92(33.0) | 191(34.2) |  |  |
| Creatinine≥2.0 mg/dL (177μmol/L) | 28(10.1) | 26(9.3) | 54(9.7) |  |  |
| Lactate dehydrogenase>upper limit of normal (>190 U/L ) | 87(31.1) | 81(29.0) | 168(30.1) |  |  |
| Albumin <35 g/ L | 45(16.1) | 32(11.5) | 77(13.8) |  |  |
